# Anti-Neoplastic Effects of Gallic Acid, a Major Component of *Toona sinensis* Leaf Extract, on Oral Squamous Carcinoma Cells

**DOI:** 10.3390/molecules15118377

**Published:** 2010-11-16

**Authors:** Yi-Chen Chia, Ranjan Rajbanshi, Colonya Calhoun, Robert H. Chiu

**Affiliations:** 1 Department of Food Science and Technology, Ta-Jen University, Ping Tung Hsien, Taiwan; 2 Dental Research Institute, UCLA School of Dentistry, Los Angeles, CA 90095, USA; 3 Department of Surgery/Oncology, David Geffen School of Medicine at UCLA, Los Angeles, CA 90095, USA; 4 Jonsson Comprehensive Cancer Center, UCLA, Los Angeles, CA 90095, USA

**Keywords:** apoptosis, necrosis, *Toona sinensis*, human oral squamous cell carcinoma, normal human oral kerationcytes, gallic acid

## Abstract

Extract of *Toona sinensis* (*TS*) has been reported to have various effects on cultured cell lines, including anti-proliferative activity in cancer cells. We have studied the effects of *TS* on various human oral squamous carcinoma cell lines (HOSCC), including UM1, UM2, SCC-4, and SCC-9. These cell lines were treated with *TS* leaf extract and screened for viability, apoptosis, necrosis, and apoptotic gene expression. Normal human oral keratinocytes (NHOK) served as a control for cytotoxic assays. Viability of *TS*-treated HOSCC was reduced, whereas that of NHOK was not affected. FACScan analysis revealed that the leaf extract induced apoptosis or a combination of apoptosis and necrosis, depending on cell type. Microarray and semi-quantitative RT-PCR analysis for apoptotic-related gene expression revealed that 3,4,5-trihydroxybenzoic acid (gallic acid, one of the major bioactive compounds purified from *TS* extract) up-regulated pro-apoptotic genes such TNF-α, TP53BP2, and GADD45A, and down-regulated the anti-apoptotic genes Survivin and cIAP1, resulting in cell death. This study suggests that gallic acid, the major bioactive compound present, is responsible for the anti-neoplastic effect of *Toona sinensis* leaf extract.

## 1. Introduction

*Toona sinensis* Roemor (*TS*), also known as *Cedrela sinensis* (class Magnoliopsida, Meliaceae family) is found widely distributed in the southeastern regions of Asia, including India, Nepal, China, Taiwan, Thailand, Malaysia, and Java [1]. It has been traditionally used as herbal medicine for as a febrifuge, astringent and carminative and as a treatment for enteritis [1]. It has recently been reported that *TS* leaf extract exerts pleiotropic biological functions such as a lypolytic effect on adipocytes [2], protective effects against hydrogen peroxide-induced oxidative stress on DNA [3], an enhancing effect on glucose uptake in 3T3-L1 adipocytes [4], and an inhibitory effect on SARS coronavirus replication [5] and leydig cell steroidogenesis [6]. In addition, the crude water extract of *TS* leaves has been demonstrated to possess anti-proliferative effects, to promoted apoptosis in human non-small lung cancer cells [7,8,9,10], and alleviate liver fibrosis [11] and hyperglycemia by altering the adipose glucose transporter 4 [12]. Most recently, the fractionated *TS* leaf extract (TSL2) was shown to induce apoptosis of human ovarian cancer cells and inhibit tumor growth in a xenograft model [13]. Gallic acid, a phenolic component purified from *TS* leaf extract, induces cell death in the human premyelocytic leukemia cell line, HL-60 [14], and in human prostate cancer cells [15]. The biologically active compound in *TS* leaf extracts may be similar to other naturally obtained compounds such as genistein, phenolic compounds, flavinoids, tea polyphenols, alkaloids, polysaccharides and glycoproteins, and lectins and terpinoids, which are known to induce apoptotic cell death in cancer cell lines via various pathways [16,17].

Fifteen known compounds were identified and isolated from *TS*, including methyl gallate, gallic acid, kaempferol, quercitin, quercitrin, catechin, epicatechin, oleic acid, palmitic acid, linoleic acid, linolenic acid, a mixture of β-sitosterol and stigmasterol, and β-sitosteryl-glucoside [18]. Of these compounds, methyl gallate has demonstrated anti‑oxidant and lipid peroxidation inhibitory activities [3], while gallic acid demonstrates antioxidant, anti-allergic, anti-inflammatory, anti-mutagenic, anti-carcinogenic, and apoptotic activities [13,19,20,21,22]. Although anti-cancer properties of gallic acid have been demonstrated [23,24], its molecular mechanism and anti-neoplastic effects on oral squamous carcinomas have not yet been explored.

Human oral squamous cell carcinoma (HOSCC) accounts for 94% of all oral malignancies. The current modalities of treatment for HOSCC include surgery, radiation, and chemotherapy, and combinations thereof, depending upon tumor type/size, lymph node involvement, and metastasis staging. The major drawbacks of currently available chemotherapeutic agents are their toxicity and non‑specific nature [25]. Therefore, improving the efficacy and safety of these therapies has become an urgent concern for both clinical oncologists and basic scientists.

## 2. Results and Discussion

### 2.1. Toona sinensis leaf extract (TSL-1) has inhibitory effects on oral squamous cell carcinoma

**Figure 1:**
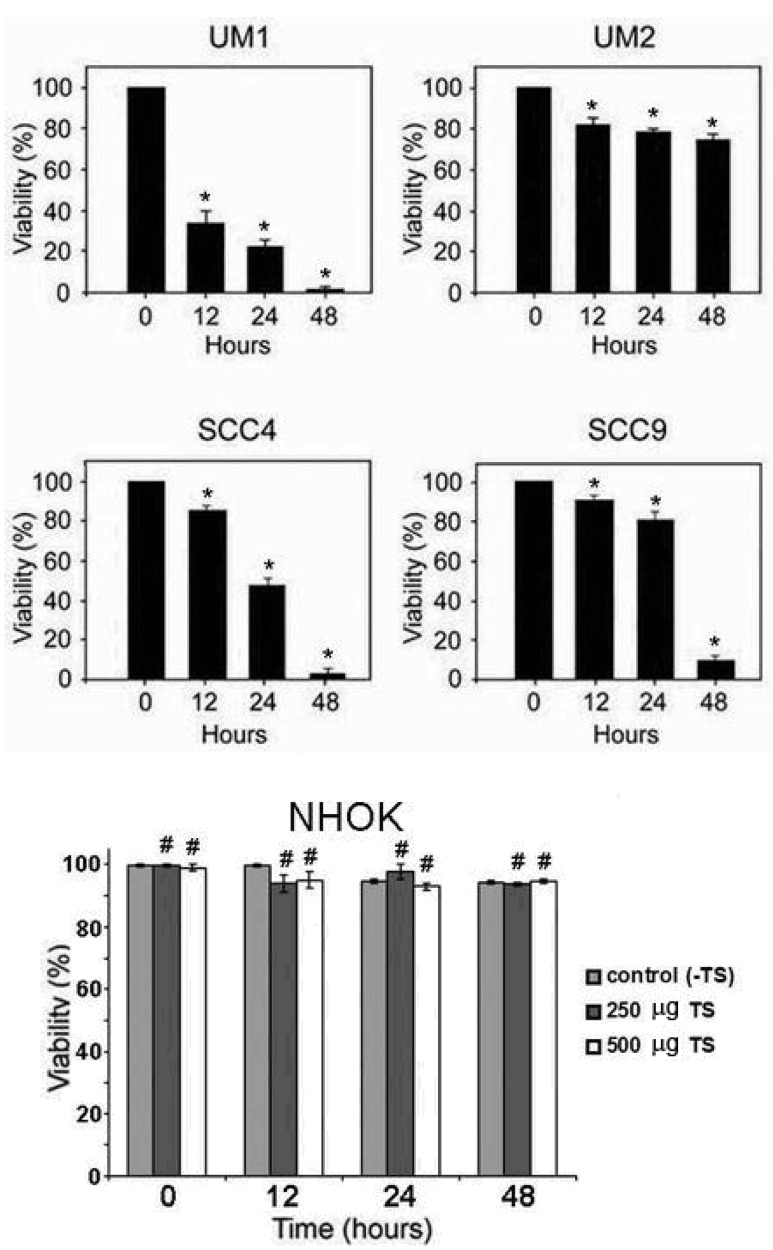
Effect of *TS* on viability of human oral squamous cell carcinoma (HOSCC) and normal human oral keratinocytes (NHOK). UM1, UM2, SCC4, and SCC9 cell lines were treated with 500 μg/mL of TSL-1 for 12, 24 or 48 hours. At the indicated time points, cells were harvested for viability assays, using trypan blue exclusion. Results are presented as mean ± SE of three independent assays (*bars*, SE; *, *P* < 0.05 compared with untreated cells). NHOK cells were treated or untreated with 250 or 500 µg/mL for 12, 24, or 48 hours. At the indicated time points, cells were harvested for viability assays. *Columns*, mean of the percentage of viability of cells from three independent experiment; *bars*, SE; #, *P* ≥ 0.05 compared with untreated NHOK control cells.

To elucidate the inhibitory effects of the *TS* leaf extract (TSL-1) on the viability of human oral squamous cell carcinoma (HOSCC), UM1, UM2, SCC-4, and SCC-9 cells were treated with 500 µg/mL of TSL-1 for various time periods. At the indicated time points, cells were harvested for viability assays. As shown in Figure 1, TSL-1 effectively caused cell death at 48 hours in SCC-4, SCC-9, and UM1 cells, whereas UM2 was less affected. To confirm the toxicity of TSL-1, primary cultured normal human oral keratinocytes (NHOK) were also subjected to 12 to 48 hours of treatment. Our results clearly demonstrated that there was no significant inhibitory effect on NHOK at indicated various time points post-treatment with 250 or 500 µg/mL of TSL-1, as compared to untreated cells (Figure 1). Thus, the inhibitory effect of TSL-1 is specific for HOSCC, but does not cause significant cytotoxicity in NHOK.

### 2.2. TSL-1 induces apoptosis in HOSCC

**Figure 2:**
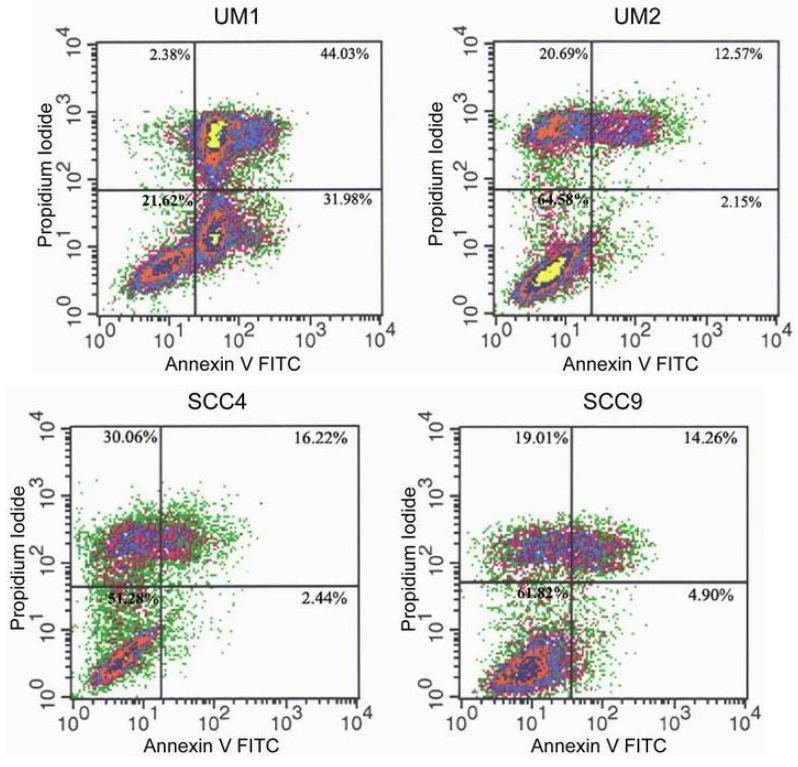
Flow cytometric analysis of *TS*-induced HOSCC cell death. UM1, UM2, SCC4, and SCC9 cells were grown in the absence (control) or the presence of TSL-1 (500 μg/mL) for 24 hours, stained with annexin V and propidium iodide, and analyzed by flow cytometry. The distributions of cells are illustrated in dot plots.

There is increasing evidence that apoptosis plays a critical role in responses to chemotherapeutic agents. To determine whether TSL-1-induced cell death is due to apoptosis, FACScan analysis was performed, using annexin V FITC and propidium iodide. Results revealed that UM1 cell death was induced primarily by apoptosis (97%), whereas UM2, SCC-4, and SCC-9 cell death was induced by late apoptosis and/or necrosis (Figure 2).

### 2.3. 3 4,5-Trihydroxybenzoic acid (gallic acid) is a major bioactive component of TSL-1

Induced HOSCC cell death indicates that TSL-1 contains bioactive compound(s) exerting anti-tumor effects. To identify the bioactive compound(s) present in TSL-1, it was further purified by HPLC separation, followed by silica gel chromatography.

**Figure 3:**
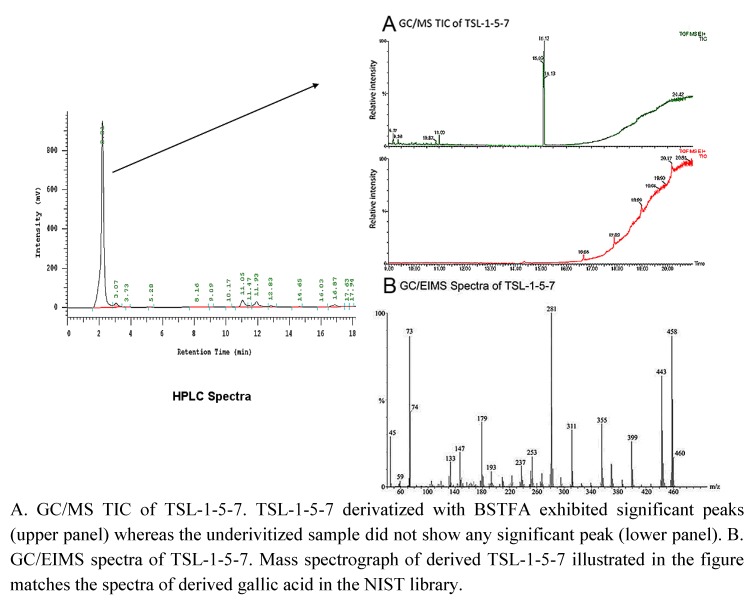
Spectra of TSL-1-5-7.

One of the fractions, TSL-1-5-7 (Figure 3, left panel), which retains anti-proliferative activity, was subjected to GC/EIMS at the Parslow Mass Spectrometry Laboratory at UCLA. The sample was derivatized for GC/EIMS analysis with bis-trimethylsilyltrifluoroacetamide (BSTFA). The total ion chromatograph profile revealed a single strong peak (Figure 3A), indicating the presence of a single major compound in the fraction. The gas chromatography electron ion spray time of flight mass spectrograph (GCT) (Figure 3B) of the compound matched the spectrograph of 3,4,5-trihydroxy benzoic acid (gallic acid) in the National Institute of Standards and Technology (NIST) library. Thus, we conclude that TSL-1 contains the bioactive compound gallic acid.

### 2.4. The presence of gallic acid in TSL-1 exerts anti-tumor activity

**Figure 4:**
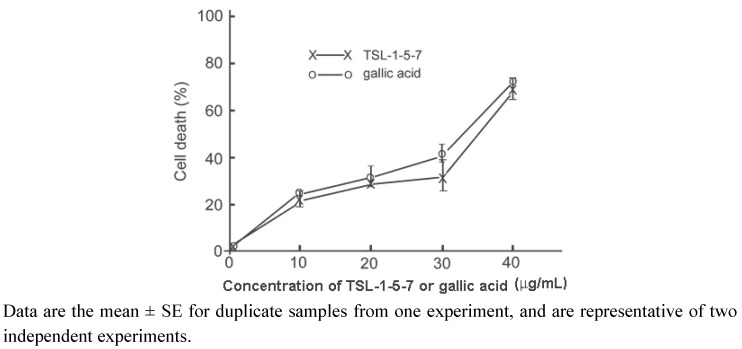
Relative potency of TSL-1-5-7- and gallic acid-induced UM1 cell death. UM1 were grown in the absence or presence of various concentrations of TSL-1-5-7 or gallic acid for 24 hours, and cell death was assessed by staining with annexin V-FITC and propidium iodide (PI), followed by flow cytometry analysis.

The gallic acid present in *TS* leaf extract was reported to exhibit anti-proliferative activity in metastatic cell lines [26]. To further confirm that the anti-neoplastic activity of TSL-1-5-7 is due to gallic acid, we performed proliferation assays in the UM1 cell line treated with various concentrations of gallic acid or TSL-1-5-7. The results revealed that the IC_50_ values of gallic acid and TSL-1-5-7 for 24 hours in UM1 cells were comparable (26.13 µg/mL in TSL-1-5-7 vs. 19.47 µg/mL in gallic acid). This was further corroborated by FACScan analysis, which demonstrated that TSL1-5-7 and gallic acid had similar potency in inducing UM1 cell death (Figure 4). We therefore conclude that gallic acid is one of the major bioactive compounds in TSL-1 that is responsible for its anti-neoplastic activity.

### 2.5. TSL-1-5-7 and gallic acid both up-regulate pro-apoptotic genes and down-regulate anti-apoptotic genes

**Figure 5:**
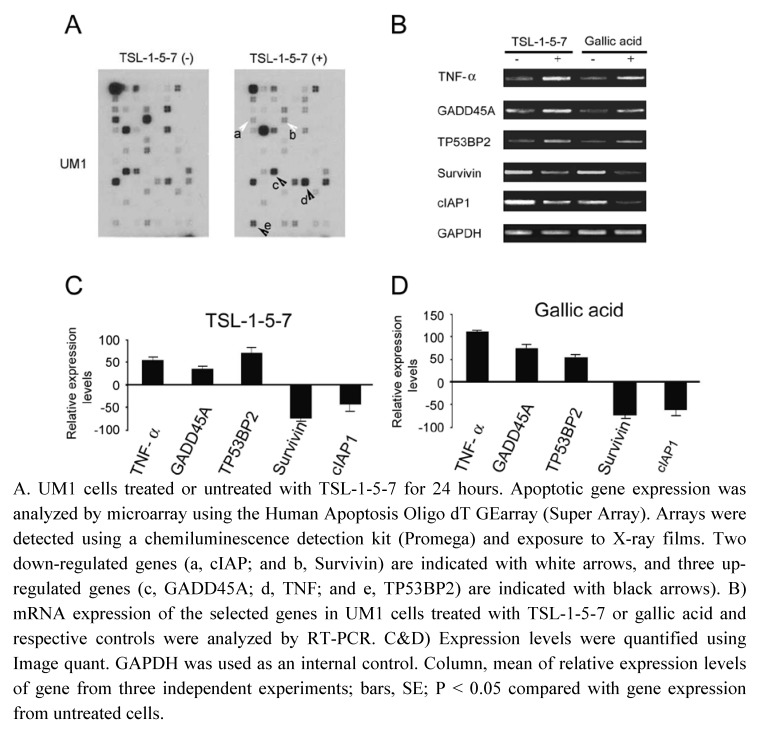
Microarray and semi-quantitative RT-PCR analysis of TNF-α, GADD45A, TP53BP2, Survivin, and cIAP expression in TSL-1-5-7- or gallic acid-treated or -untreated UM1 cells.

To examine the effect of TSL-1 in inducing cell death by altering expression of apoptotic genes, we performed microarray analysis with mRNA isolated from TSL-1-5-7-treated-UM1 cells, and compared them to the mRNA probe isolated from untreated cells. We found alterations in expression of apoptotic genes in UM1 cell lines treated with TSL-1-5-7 when compared to their controls (Figure 5A). To examine similarity of gallic acid to TSL-1 in affecting apoptotic gene expression, semi-quantitative RT-PCR was performed for up-regulated genes such as TNF-α, TP53BP2, and GADD45A, and for down-regulated genes, Survivin and cIAP1, in UM-1 cells treated with TSL‑1-5-7 or gallic acid, compared to their respective controls (Figure 5B). PCR amplification signals were quantified using Image Quant Software (Figure 5C and D). A similar up-regulated and down-regulated pattern of apoptotic associated genes by treatment of TSL-1-5-7 and gallic a acid are shown in Figure 5C and D, suggesting gallic acid has a similar or same function in regulation of pro-apoptotic and anti-apoptotic gene expression.

### 2.6. Discussion

We investigated the effects of the potential chemotherapeutic herbal product in a *TS* leaf extract, TSL-1, which contains the bioactive compound gallic acid, to selectively inhibit HOSCC cell viability, but not affect normal control cells *in vitro*. TSL-1 could be more effective in a highly metastatic cell line, as UM1 exhibits higher metastatic potential than UM2 [27]. To determine whether the anti-proliferative effects of TSL-1 on HOSCC are due to apoptosis or a combination of apoptosis and necrosis, we performed a FACScan analysis. Our results clearly demonstrated that TSL-1 induces HOSCC cell death by either apoptosis or a combination of apoptosis and necrosis. These differing effects could be due to cell type-dependent reaction after treatment with *TS* leaf extract. The mechanism for the apoptotic and/or necrotic cell death observed in UM2, SCC4, and SCC9 cells, remain to be elucidated.

It is known that apoptotic pathways are deregulated in cancer [28], so induction of apoptotic and/or necrotic cell death in oral cancer cell lines by *TS* leaf extracts shows promise as an anti‑neoplastic therapy. Using microarray analysis, we observed that the pro-apoptotic genes, TNF-α, TP53BP2, and GADD45A, are up-regulated, while the anti-apoptotic genes, survivin and cIAP1, are down-regulated in UM1 cells treated with TS leaf extract fractionated TSL-1-5-7 or gallic acid. Up-regulation of TNF-α in UM1 cells suggests that both extrinsic [29] and intrinsic pathways of apoptosis [30] are involved in *TS*-induced cell death. TNF-α is one of the prime signals that induces apoptosis in a variety cells. Conversely, it also activates the transcription factor, NFκB, which has a protective role against apoptosis induced by TNF-α, ionizing radiation, and chemotherapeutic agents such as doxorubicin [31]. Up‑regulated TP53BP2 and GADD45A could induce apoptosis through the mitochondria death pathway [32,33], but the underlying mechanism remains to be elucidated.

Down-regulation of Survivin (BIRC5) in TSL-1-5-7- or gallic acid-treated UM1 cells suggests the possibility of dysregulated mitotic progression and triggering of tumor cell apoptosis. Cell death induced by Survivin targeting exhibited the hallmarks of mitochondrial-dependent apoptosis, with release of cytochrome C and loss of mitochondrial transmembrane potential [34]. Similarly, cIAP1 down-regulation suggests activation of caspase 9 [35,36], which leads to activation of caspase-3 and triggers tumor cell apoptosis. *TS* fraction TSL-1-5-7- and gallic acid-induced apoptosis was associated with up-regulation of pro-apoptotic genes and down-regulation of anti-apoptotic genes, leading to apoptosis of UM1 cells. These data are in agreement with a previous report that gallic acid up-regulates Bcl2 and down-regulates Bax in gallic acid-treated HL-60 cells, and provides evidence that *TS*-induced cell death is apoptotic [14]. Recently, gallic acid was found to block growth of the DU145 prostate cancer cell at the G2/M phases of the cell cycle by activation of Chk1 and Chk2 and inhibition of Cdc25C and Cdc2 activity [15].

3,4,5-Trihydroxybenzoic acid (gallic acid) was identified as one of the major bioactive compounds present in *TS* leaf extract in this study. Gallic acid has been reported to cause cell death in lung cancer and other tumor cells [3,26,37]. It has antioxidant properties and has been commonly used as an antioxidant additive in high-fat foods and in stored medicinal preparations [38]. Other reports have shown that intracellular gallic acid induces ROS, especially H_2_O_2_, which plays an important role in eliciting an early signal in apoptosis [24,39]. Our results indicate that the major bioactive component, gallic acid, induces UM1 cell death by apoptosis. Gallic acid has been demonstrated to act synergistically with doxorubicin to suppress growth of DU145 cells [15]. Recently, there is increasing evidence that many naturally isolated compounds and Chinese medicinal herbs offer promising biological modifiers applicable to cancer treatment. For example, oral administration of gallic acid was shown to suppress the growth of transplanted lung cancer by inducing tumor cell apoptosis *in vivo*, and to significantly enhance the efficacy of cisplatin in inducing apoptosis and suppressing tumor growth [40].

## 3. Experimental

### 3.1. Cell culture and chemical reagents

The oral squamous cell carcinoma cell lines, UM1 and UM2, were obtained from Xiaofeng Zhou (UCLA School of Dentistry), and the SCC-4 and SCC-9 cell lines were purchased from the American Type Culture Collection (ATCC). UM1 and UM2 cells were cultured in DMEM/F12, and SCC-4 and SCC-9 cell lines in DMEM/F12 supplemented with 400 ng/mL hydrocortisone. Normal human oral keratinocytes (NHOK) were cultured in keratinocyte basal media containing keratinocyte growth factor. All media contained 10% heat-inactivated fetal bovine serum and antibiotics. Cell cultures were grown in a humidified atmosphere of 5% CO2, 95% air at 37 °C. Trypan blue, hydrocortisone, and gallic acid were obtained from MP Biomedicals, Cal Biochem Inc., and Sigma Aldrich Inc., respectively.

### 3.2. Toona sinensis leaf extract preparation

Aqueous crude extract of *TS* from leaves was obtained by boiling 100 grams of leaves in 1,000 mL of water until only 100 mL remained. The aqueous crude extract was centrifuged at 3,000 rpm for 12 minutes, and the supernatant was lyophilized to obtain TSL-1. A purified fraction, TSL1-5-7, was obtained by HPLC separation of TSL-1, followed by silica gel chromatography of the HPLC TSL-1-5 fraction.

### 3.3. Viability and IC_50_ assays

Viability assays were performed using 0.4% trypan blue, as described previously [41]. Briefly, TSL-1-treated and control cells were harvested and resuspended in Hank’s balanced salt solution and 0.4% trypan blue. Cells were incubated at room temperature for 5-10 minutes before counting. Cell viability was assayed based upon trypan blue exclusion, and visualized under a light microscope on a Neubauer’s improved haemocytometer. Assays for determination of 50% inhibition concentration (IC_50_) for proliferation of UM1 by TSL-1-5-7 and gallic acid for 24 hours were performed as described previously [42], using Sigma plot 9.1.

### 3.4. Flow cytometry analysis

The Apoalert kit (Becton-Dickinson) was used to stain cells with annexin V-FITC and/or propidium iodide according to the manufacturer’s protocol. Approximately 10,000 cells were analysed from each sample. Flow cytometric analysis was performed by FACScan (Becton-Dickinson). Apoptotic and/or necrotic cells were identified by staining with annexin V-FITC and propidium iodide. The percentages of distribution of normal (Annexin V-FITC-/PI-), early apoptotic (Annexin V-FITC+/PI-), late apoptotic (annexin V-FITC+/PI+) and necrotic cells (Annexin V-FITC-/PI+) were calculated by the Cell Quest software.

### 3.5. Semi-quantitative reverse transcription polymerase chain reaction (RT-PCR)

Total RNA was reverse transcribed to obtain cDNA, using Superscript III (Invitrogen) according to the manufacturer’s protocol. Primers for all the genes of interest were obtained from Sigma Genosys. Initial denaturation for PCR occurred at 95 °C for 3 minutes, and further cycles of denaturation, annealing, and elongation were performed at 95 °C, 57 °C, and 72 °C, respectively, for 30 seconds. GAPDH, a housekeeping gene, served as the internal control. The PCR products were analyzed using 2% agarose gels, and the resolved bands were quantified using Image Quant 5.0.

### 3.6. GC/EIMS analysis

TSL-1-5-7 was dissolved in acetonitrile and analyzed by a gas chromatograph (Agilent Technologies model 6890) and an API III+ triple quadrupole mass spectrometer (Perkin-Elmer Sciex). A capillary GC column (60 m × 0.26 mm ID × 0.25 μm film thickness) was interfaced directly with the mass spectrometry (MS) source. The oven temperature was held at 50°C for three minutes, and subsequently programmed to rise to 350 °C at increments of 20 °C/min. MS conditions were ion source 180 °C and electron energy 70 eV. Helium served as the carrier gas. The MassLynx (Waters) matching algorithm and library of spectra from the National Institute of Science and Technology (NIST 05) were used to identify the compound.

### 3.7. Statistical analysis

Results are expressed as means ± SE from at least three independent experiments. Statistical analysis was performed using student’s t test. Unless otherwise indicated, *P* < 0.05 was deemed significant.

## 4. Conclusions

We have demonstrated that *TS* leaf extract induces cell death in oral squamous cell carcinoma cells but not in normal oral epithelial cells. Flow cytometry analysis revealed that *TS* induces cell death via apoptosis in the UM1 cell line, and via late apoptosis and/or necrosis in the UM2, SCC4, and SCC9 cell lines. Furthermore, gallic acid was identified as the major bioactive compound present in *TS* leaf extract, TSL-1, which induces anti-neoplastic activity by up-regulating pro-apoptotic genes and down-regulating anti-apoptotic genes, resulting in cell death.
